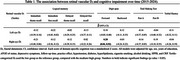# Association of retinal vascular fractal dimension with cognitive impairment among community‐dwelling older adults in Taiwan

**DOI:** 10.1002/alz70856_100694

**Published:** 2025-12-24

**Authors:** Hsin‐Jou Wang, Yi‐Ting Hsieh, Ting‐Yu Wu, Jeng‐Min Chiou, Jen‐Hau Chen, Yen‐Ching Chen

**Affiliations:** ^1^ Institute of Epidemiology and Preventive Medicine, College of Public Health, National Taiwan University, Taipei, Taiwan; ^2^ National Taiwan University Hospital, Taipei, Taiwan; ^3^ Taipei Medical University‐Shuang Ho Hospital, Ministry of Health and Welfare, New Taipei, Taiwan; ^4^ Institute of Statistics and Data Science, National Taiwan University, Taipei, Taiwan; ^5^ Institute of Statistical Science, Academia Sinica, Taipei, Taiwan; ^6^ National Taiwan University Hospital Yunlin Branch, Yunlin, Taiwan; ^7^ College of Medicine, National Taiwan University, Taipei, Taiwan

## Abstract

**Background:**

The eye's vascular system, sharing developmental origins and high metabolic demands with the brain, is an essential early predictor of cognitive impairment. This study investigates the association between retinal vascular fractal dimension (D_f_) and cognitive function, addressing gaps in longitudinal research with a comprehensive domain‐specific assessment in community‐dwelling older adults.

**Method:**

Data (2015‐2024) were drawn from the Taiwan Initiative for Geriatric Epidemiological Research (TIGER), an ongoing prospective cohort study initiated in 2011. Between 2015 and 2017 (baseline of this study), 276 older adults aged 65 or older were included after applying exclusion criteria in this study. Retinal images were captured using a Canon CR‐2 AF fundus camera. Retinal vascular complexity was quantified using the box‐counting method to calculate D_f_, with lower D_f_ values indicating reduced branching complexity. Global cognition was assessed biennially with four repeats using the Montreal Cognitive Assessment‐Taiwanese version and across four domains (memory, attention, verbal fluency, and executive function) using a battery of neuropsychological tests. Generalized linear mixed models examined associations between tertile‐categorized D_f_ and cognition, adjusting for age, sex, years of education, apolipoprotein E *(APOE)* ε4 status, depressive symptoms, and other covariates.

**Result:**

The mean age of participants was 75.3 years old with 56.2% of women. The distributions of hyperlipidemia, age, and short physical performance battery (SPPB) scores significantly differed between two groups (low vs. medium+high) of the retinal vascular dimension of the right eye (*p*‐value=0.003‐0.04). In contrast, significant differences were found in hypertension, age, SPPB scores, and C‐reactive protein (CRP) for the left eye (*p*‐value=<.0001‐0.04). A significant association was found between right eye D_f_ (low vs. medium+high) and performance in the attention domain (β=0.28), which was particularly evident in women, younger individuals, and those with higher education levels. No significant associations were found in other cognitive domains or while treating D_f_ as a continuous variable.

**Conclusion:**

Over time, we found that higher retinal vascular D_f_ was associated with better attention performance in the community‐dwelling elders in the TIGER cohort, especially for the right eye. Further research is required to validate these findings and clarify the mechanisms linking eye vascular complexity differences to cognitive impairment.